# Supplementary material for: Species of Dickeya and Pectobacterium Isolated during an Outbreak of Blackleg and Soft Rot of Potato in Northeastern and North Central United States
Source: Microorganisms. 2021 Aug 14;9(8):1733. doi: 10.3390/microorganisms9081733 (PMC8401272; doi:10.3390/microorganisms9081733)
Supplement: Supplementary file 1 [file microorganisms-09-01733-s001.zip › microorganisms-1293507-supplementary.pdf]

Table S1. Haplotypes of dnaJ, dnaX, and gyrB and sequence types (ST) of strains within the MLSA clade of *Dickeya dianthicola*.

Strain[a]	Geographic origin	haplotypes[b] *dnaJ*	*dnaX*	*gyrB*	ST[c]
FL13	Florida	1	1	1	1
16MB-01	Maine	1	1	1	1
ME23	Maine	1	1	1	1
ME30	Maine	1	1	1	1
ST64	Maine	1	1	1	1
16MA-15T	Massachusetts	1	1	1	1
2820	Michigan	1	1	1	1
NY1528B	New York	1	1	1	1
NY1536B	New York	1	1	1	1
NY1547B	New York	1	1	1	1
NY1557A	New York	1	1	1	1
NY1558D	New York	1	1	1	1
NY1559C	New York	1	1	1	1
NY1562C	New York	1	1	1	1
NY1578A	New York	1	1	1	1
BP7034	Pennsylvania	1	1	1	1
PA24	Pennsylvania	1	1	1	1
NY1556C	New York	2	1	1	2
NCPPB 3534	Netherlands	3	1	2	3
IPO 980	Netherlands	4	1	3	4
RNS04.9	France	1	2	3	5
600	Georgia	1	2	3	5
16NJ-12 1	New Jersey	1	2	3	5
LMG 2485[T]	United Kingdom	1	2	3	5
GBBC 2039	Belgium	4	2	3	6

[a] All strains from Florida, Maine, Massachusetts, Michigan, New Jersey, New York, and Pennsylvania originated from potato or water samples collected in 2015 and 2016. Strain 600 is a historical strain isolated previously from sweet potato in Georgia (date unknown) and held in the R. S. Dickey collection. LMG 2485T was obtained from the Belgian Co-Ordinated Collections of Micro-Organisms (BCCM/LMG).

[b] Sequence data for reference strains NCPPB 3534, IPO 980, GBBC 2039, and RNS04.9 were obtained from GenBank and ASAP, as described in Table 3. All other DNA sequences were amplified in this study. Sequences of dnaJ (672 bp), dnaX (450 bp), and gyrB (822 bp) having at least a single nucleotide difference were assigned different haplotypes for that locus.

[c] Each unique concatenated sequence, based on allelic variation of dnaJ, dnaX, and gyrB, was assigned a unique sequence type (ST).

Table S2. Allelic variation in dnaJ, dnaX, and gyrB and sequence types (ST) of strains within the MLSA clade of *Pectobacterium parmentieri*.

Strain[a]	Geographic origin	***dnaJ***	*dnaX*	**haplotypes[b]** *gyrB*	ST[c]
3230	Michigan	4	1	4	1
RNS 08-42-1A[T]	France	7	1	1	2
CIR1146	Minnesota	7	1	1	2
CIR1018	Minnesota	7	1	1	2
CIR1056	Minnesota	7	1	1	2
CIR1058	Minnesota	7	1	1	2
CIR1059	Minnesota	7	1	1	2
CIR1009	Minnesota	7	1	1	2
NY1532B	New York	7	1	1	2
CIR1102	North Dakota	7	1	1	2

CIR1114	North Dakota	7	1	1	2
CIR1127	North Dakota	7	1	1	2
CIR1137	North Dakota	7	1	1	2
CIR1160	North Dakota	7	1	1	2
CIR1095	North Dakota	7	1	1	2
NY1533B	New York	6	3	5	3
NY1539A	New York	1	3	4	4
Scc3193	Finland	1	2	6	5
CIR1175	Minnesota	1	2	6	5
CIR1176	Minnesota	1	2	6	5
CIR1177	Minnesota	1	2	6	5
CIR1178	Minnesota	1	2	6	5
CIR1179	Minnesota	1	2	6	5
CIR1180	Minnesota	1	2	6	5
CIR1181	Minnesota	1	2	6	5
NY1548A	New York	1	2	6	5
NY1585A	New York	1	2	6	5
NY1587A	New York	1	2	6	5
CIR1054	North Dakota	1	2	6	5
CIR1055	North Dakota	1	2	6	5
NY1584A	New York	1	3	5	6
NY1588A	New York	2	2	6	7
CIR1153	Minnesota	3	1	2	8
CIR1154	Minnesota	3	1	2	8
CIR1002	Minnesota	3	1	2	8
CIR1108	North Dakota	3	1	2	8
CIR1019	Minnesota	7	4	1	9
CIR1021	Minnesota	5	2	3	10
CIR1051	North Dakota	5	2	3	10

[a] All strains from Michigan, Minnesota, New York, and North Dakota originated from potato samples collected in 2015 and 2016.

[b] Sequence data for the type strain RNS 08-42-1AT and for reference strain Scc3193 were obtained from GenBank and ASAP, as described in Tables 2 and 3. All other DNA sequences were amplified in this study. Sequences of dnaJ (672 bp), dnaX (450 bp), and gyrB (711 bp) having at least one nucleotide difference were assigned different haplotype numbers.

[c] Each unique concatenated sequence, based on haplotypes of dnaJ, dnaX, and gyrB, was assigned a unique sequence type (ST).
